# Brain Gray Matter Alterations in Hepatic Encephalopathy: A Voxel-Based Meta-Analysis of Whole-Brain Studies

**DOI:** 10.3389/fnhum.2022.838666

**Published:** 2022-04-18

**Authors:** Licheng Zhu, Weihua Zhang, Lei Chen, Yanqiao Ren, Yanyan Cao, Tao Sun, Bo Sun, Jia Liu, Jing Wang, Chuansheng Zheng

**Affiliations:** ^1^Department of Radiology, Union Hospital, Tongji Medical College, Huazhong University of Science and Technology, Wuhan, China; ^2^Hubei Province Key Laboratory of Molecular Imaging, Wuhan, China

**Keywords:** seed-based d mapping, voxel-based morphometry, meta-analysis, hepatic encephalopathy, gray matter, plasma ammonia

## Abstract

**Background:**

Previous studies on voxel-based morphometry (VBM) have found that there were gray matter alterations in patients with hepatic encephalopathy (HE). However, the reported results were inconsistent and lack a quantitative review. Therefore, this study aims for a quantitative meta-analysis of VBM analysis on patients with HE.

**Methods:**

The studies in our meta-analysis were collected from Pubmed, Web of Science, and Embase, which were published from January 1947 to October 2021. The seed-based d mapping (SDM) method was applied to quantitatively estimate the regional gray matter abnormalities in patients with HE. A meta-regression analysis was applied to evaluate the relationship between plasma ammonia and gray matter alteration.

**Results:**

There were nine studies, with sixteen datasets consisting of 333 participants with HE and 429 healthy controls. The pooled and subgroup meta-analyses showed an increase in gray matter volume (GMV) in the bilateral thalamus and the calcarine fissure but a decrease in the GMV in the bilateral insula, the basal ganglia, the anterior cingulate gyrus, and the cerebellum. The meta-regression showed that plasma ammonia was positively associated with the GMV in the left thalamus but was negatively associated with the GMV in the cerebellum and the bilateral striatum.

**Conclusion:**

Gray matter volume in patients with HE largely varied and could be affected by plasma ammonia. The findings of this study could help us to better understand the pathophysiology of cognitive dysfunction in patients with HE.

## Introduction

Hepatic encephalopathy (HE) is a neuropsychiatric syndrome, which is featured by a range of clinical manifestations, such as psychometric changes, numbness, and coma. Minimal HE (MHE) is a subset of HE in which symptoms are undetectable by routine examination and can only be diagnosed by neuropsychological measurements and neurophysiological tests (Tao et al., 2013). As reported, MHE can reduce the quality of life and the working ability (Bajaj et al., 2009), and MHE is prone to progress to overt HE (OHE) without proper treatment. Patients with cirrhosis with OHE usually develop poor prognosis and a loss of learning ability (Umapathy et al., 2014). Thus, even patients recovering from the disease can still have abnormal neuropsychiatric function and high recurrence rates (Sharma et al., 2010).

The voxel-based morphometry (VBM) method has many advantages over the region of interest (ROI) analysis ability (Pereira et al., 2010), such as automation, assumption-free, operator independence, and whole-brain gray matter abnormalities. With VBM, MRI morphometry studies have focused on structural abnormalities of gray matter and white matter. However, the structures to be evaluated need to be determined in advance, because of the major limitations of ROI-based brain morphological change measurement techniques (Busatto et al., 2008). Because of the different sample sizes of participants and different research methods, the results of these studies were contradicted (Costafreda et al., 2009). In comparison, VBM is a more accurate method than the manual volume approach and can overcome the limitations of the ROI approach. It is important to confirm unanimous results of VBM studies on gray matter volume (GMV) in those with HE based on a meta-analysis (Wang et al., 2015).

At present, several VBM studies of GMV in HE have been published. However, the results are diverse. For instance, one study found that regional gray matter atrophy was mostly confined in bilateral frontal regions, the bilateral temporal pole, and the cerebellum (García-García et al., 2017), while another study suggested that the loss of gray matter was observed in the precuneus, the bilateral insular cortex, and the caudate nucleus (Chen et al., 2012).

Seed-based d mapping (SDM) is a statistical software for meta-analysis, using neuroimaging technology to detect differences in brain activity. The SDM, combining positive and negative differences, has proven to be better than methods such as the activation likelihood estimation and the multilevel kernel density analysis (Radua et al., 2012). In addition, to assess the robustness and heterogeneity of the results, SDM enables several complementary analyses, such as jack-knife and subgroup analysis (Radua et al., 2012). However, SDM has not been applied to a meta-analysis comparing patients with HE with normal controls. In this study, we have used SDM to evaluate the published VBM studies on patients with HE and the control group for identifying consistent regional gray matter abnormalities.

## Methods

### Search Strategy

Our study followed the Preferred Reporting Items for Systematic Reviews and Meta-Analyses (PRISMA) guidelines (Liberati et al., 2009). The studies in our meta-analysis were collected from Pubmed, Web of Science, and Embase, which were published from January 1947 to October 2021. The last search was run on October 25, 2021. The keywords we used were “hepatopathy” or “cirrhosis” or “hepatic fibrosis” or “HE” plus “VBM” or “voxel-based morphometry” or “gray matter” or “voxel-wise” or “voxel-based.” In addition, we also manually checked the reference lists of the included studies.

At the same time, manual retrieval was performed on the bibliography of the included literature. The inclusion criteria were as follows: (1) the studies that reported a VBM (GM volume) comparison between patients with HE and controls; (2) the results of the stereotactic changes of the whole brain’s three-dimensional coordinates (X, Y, and Z) were reported; and (3) the significance threshold was corrected by multiple comparisons based on voxels or the threshold corrected without a spatial range. We found that some studies had many independent patient samples, which were considered separate studies in our meta-analysis. The exclusion criteria are as follows: (1) not enough data could be obtained even if the corresponding author has been asked for more information; (2) there are less than 10 cases in the HE group and the control group; (3) overlapping with data from other publications; (4) uncorrected results and spatial thresholds were not reported; (5) no control group was present; (6) the analysis was limited to a specific ROI; and (7) the coordinates were not in Talairach or the Montreal Neurological Institute (MNI) space.

### Data Extraction

Two investigators (LCZ and WHZ) independently examined abstracts from the initial search, and disagreements were discussed with a third author to reach a consensus. Authors were blinded to the articles’ authors, their institutions, and the source of funding to minimize potential bias. The full texts of studies thought to fulfill the inclusion criteria were assessed in detail to confirm eligibility. Furthermore, we found that some studies had multiple independent patient samples who were compared with the same healthy control groups, which were considered in this study as separate studies in our meta-analysis. Coordinates were extracted independently by two authors using anisotropic effect size-SDM.

### Voxel-Wise Meta-Analysis

We used the SDM to analyze the gray matter difference. First, we had a pooled meta-analysis of these selected studies. Then, we conducted 3 subgroup meta-analyses, namely, non-HE (NHE), MHE, and OHE, which were three different types of HE. The SDM method has been described clearly in other articles (Radua and Mataix-Cols, 2009; Radua et al., 2011, 2012). All the reported peak coordinates were selected. To avoid potential bias, all studies used the same statistical threshold. Second, the standard MNI plots of gray matter differences for each study were reconstructed. The peak *t*-value of Hedges’ effect size was the basis of the peak coordinates’ reconstruction. The zero findings in the study were reconstructed with the same effect size, and the only difference was that all the voxels in the effect size graph were estimated to be zero. Third, an analysis was carried out by studying the average value of maps. Thus, a study that had a larger sample size would have a larger contribution. Finally, after determining the statistical significance, a zero distribution was created and the *P*-value was obtained directly. The default SDM kernel sizes and thresholds were used (full width at half-maximum D 20 mm, voxel p D 0.005, peak height Z D 1, and cluster extent D 10 voxels) (Radua et al., 2012). In addition, the robustness of the findings was assessed by using jack-knife sensitivity. For instance, data were repeatedly analyzed fifteen times in polled meta-analyses, and whenever one study was removed, a repeat analysis was performed. If a previously important brain region remained important in most of the study combinations, the finding was supposed to be highly repetitive.

### Meta-Regression Analysis

The main output of each variable was a regression slope graph. A simple linear regression can be used to assess the impact of sociodemographic and clinical variables (Radua et al., 2012). According to the previous analyses, the detection of reduced spurious associations, with a probability threshold of 0.00005, required anomalies to be found in the slope and one extreme of explanatory variables, with discarded results found in other areas of the analysis. Finally, we examined the regression graph to exclude fitting driven by too few studies (Radua and Mataix-Cols, 2009).

## Results

### Included Studies and Sample Characteristics

**FIGURE 1:**
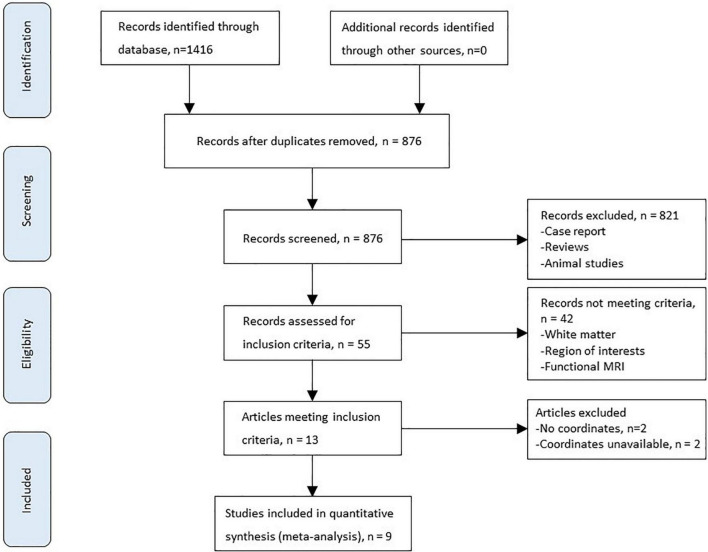
A flowchart of the inclusion and identification in the current meta-analysis.

Figure 1 shows the flowchart of inclusion and identification in this meta-analysis. In total, 1,416 documents were identified by the search strategy, and 9 (Chen et al., 2012; Iwasa et al., 2012; Zhang et al., 2012; Qi et al., 2013c; Tao et al., 2013; García-García et al., 2017; Wang et al., 2017, 2019; Liu et al., 2019) VBM studies met the inclusion criterion of comparison of GMV alterations. In five studies, the analysis was based on two or three different HE subgroups and was compared with the same healthy control group. As a result, a total of 16 groups of data were included for meta-analysis. Table 1 shows the clinical and demographical data. Table 2 shows the technical details of the included studies. All the studies included in the meta-analysis were prospective. There were 333 patients and 429 healthy controls included. No statistical difference was found in age and gender in each study.

**TABLE 1:** Demographic and clinical characteristics of studies included in the current meta-analysis.

	Patient information							Healthy controls		
Study	Types	No	Female (%)	Mean age	Child-Pugh	Comorbidity	Plasma ammonia	No	Female (%)	Mean age
				(year)	A/B/C		(μmol/L)			(year)
Zhang et al. (2012)	NHE	31	26.7	49.7	NA	Negative	58.9	40	35.0	49.8
	MHE	18	26.7	49.7	NA	Negative	58.9	40	35.0	49.8
	OHE	11	26.7	49.7	NA	Negative	58.9	40	35.0	49.8
Iwasa et al. (2012)	NHE	18	72.2	65.7	11/4/3	Negative	65.8	16	56.3	68.7
Tao et al., 2013	NHE	24	33.3	47.7	10/8/6	Negative	48.5	33	33.3	45.2
	MHE	23	26.1	44.7	4/6/13	Negative	56.5	33	33.3	45.2
	OHE	24	29.2	47.1	0/2/22	Negative	75.4	33	33.3	45.2
Wang et al. (2019)	NHE	20	45.0	56.0	4/4/7	Negative	51.6	20	30.0	51.0
Liu et al. (2019)	OHE	23	8.7	45.9	10/10/3	Negative	NA	23	13.0	51.8
	OHE	23	8.7	50.4	4/16/3	HM	NA	23	13.0	51.8
García-García et al. (2017)	NHE	26	23.1	63.0	23/3/0	Negative	NA	24	33.3	61.0
	MHE	13	7.7	64.0	8/5/0	Negative	NA	24	33.3	61.0
Wang et al. (2017)	NHE	17	29.4	53.4	15/1/1	Negative	39.0	17	29.4	54.4
	NHE	17	29.4	54.8	15/1/1	Diabetes	54.5	17	29.4	54.4
Chen et al. (2012)	OHE	20	14.2	51.9	NA	Negative	NA	21	15.0	51.2
Qi et al. (2013c)	MHE	25	28.0	56.2	12/12/1	Negative	70.1	25	28.0	53.7

*NHE, non hepatic encephalopathy; MHE, minimal hepatic encephalopathy; OHE, overt hepatic encephalopathy; NA, not available, HM, Hepatic myelopathy.*

**TABLE 2:** Technique details of VBM studies for GMV on HE in meta-analysis.

Study	Scanner (T)	Software	FHWH (mm)	*P*-value	Coordinates
Zhang et al. (2012)	3	SPM8	8	*P* < 0.01 (FDR correction)	36
Iwasa et al. (2012)	1.5	SPM8	8	*P* < 0.001 (uncorrected)	5
Tao et al. (2013)	3	SPM8	8	*P* < 0.05 (FDR correction)	22
Wang et al. (2019)	3	SPM8	3	*P* < 0.0002 (FWE correction)	7
Liu et al., 2019	3	SPM8	12	*P* < 0.05 (AlphaSim correction)	21
García-García et al. (2017)	3	SPM12	8	*P* < 0.001 (uncorrected)	49
Wang et al. (2017)	3	SPM8	8	*P* < 0.05 (Alphasim correction)	19
Chen et al. (2012)	1.5	SPM5	4	*P* < 0.05 (FDR corrected)	24
Qi et al. (2013c)	3	SPM8	8	*P* < 0.05 (FDR correction)	15

*HE, hepatic encephalopathy; FDR, false discovery rate; FEW, family-wise error; FWHM, full width at half-maximum; SPM, Statistical Parametric Mapping; T, Tesla; VBM, voxel-based morphometry; GMV, gray matter volume.*

### Pooled Meta-Analysis of All the Studies

**FIGURE 2:**
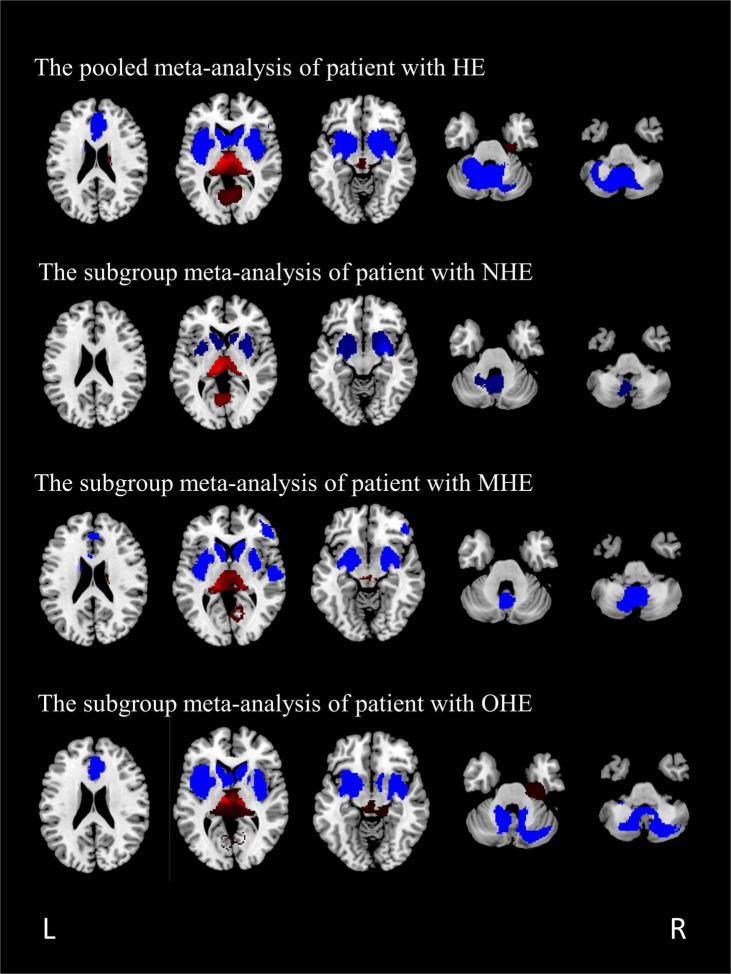
The regions of decreased (blue) and increased (red) gray matter volumes (GMVs) in patients with HE compared with healthy controls in a pooled meta-analysis and three subgroup meta-analyses. The results are displayed on a 2D axial rendered brain. HE, hepatic encephalopathy; NHE, non-hepatic encephalopathy; MHE, minimal hepatic encephalopathy; OHE, overt hepatic encephalopathy; L, left; R, right.

The GMV reduction in patients with HE was mainly found in four clusters (Table 3 and Figure 2). The largest cluster is located in the bilateral insula, which extended into the bilateral basal ganglia. The second largest cluster is located in the left cerebellar hemisphere and the cerebellar vermis. GMV also decreased in the bilateral anterior cingulate gyrus and the right inferior frontal gyrus. Meanwhile, GMV increment was observed in the bilateral thalamus, the lingual gyrus, and the calcarine fissure and its surrounding cortex in patients with HE.

**TABLE 3:** Altered gray matter volume for pooled meta-analysis of all the included studies.

	Maximum			Cluster		Jackknife sensitivity analysis (combination of studies detecting the differences)
Brain regions	MNI coordinates, x, y, z	SDM value	*P*-value	No. of voxels	Breakdown (no. of voxels)	
**HE > control**						
R thalamus	6, −20, 12	4.293	<0.001	1062	R thalamus (466)	16 out of 16
					L thalamus (447)	
					R lingual gyrus, BA 27 (40)	
					L hippocampus, BA 27 (33)	
					R hippocampus (11)	
					L lingual gyrus (8)	
R calcarine fissure / surrounding cortex	4, −64, 10	2.186	<0.001	1017	L calcarine fissure / surrounding cortex, BA 17 (211)	16 out of 16
					R lingual gyrus, BA 18 (274)	
					L lingual gyrus, BA 17 (266)	
					R calcarine fissure / surrounding cortex, BA 17 (159)	
					Cerebellum, vermic lobule IV/V (56)	
					R precuneus, BA 30 (25)	
					L precuneus, BA 17 (26)	
**HE < control**						
L insula, BA 48	−28, 12, 6	−3.764	<0.001	4344	L insula, BA 48 (973)	16 out of 16
					R striatum (574)	
					L striatum (490)	
					R lenticular nucleus, putamen, BA 48 (464)	
					R insula, BA 48 (419)	
					L lenticular nucleus, putamen, BA 48 (269)	
					R caudate nucleus (260)	
					L caudate nucleus (225)	
					R amygdala, BA 34 (194)	
					L amygdala, BA 34 (188)	
					L parahippocampal gyrus, BA 28 (114)	
					R temporal pole, superior temporal gyrus (89)	
					R parahippocampal gyrus, BA 28 (85)	
L cerebellum, hemispheric lobule	−6, −62, −40	−4.121	<0.001	3049	L cerebellum, hemispheric lobule (1853)	16 out of 16
					Cerebellum, vermic lobule (722)	
					R cerebellum, hemispheric lobule (474)	
R anterior cingulate / paracingulate gyri	10, 40, 14	−3.415	<0.001	2211	R anterior cingulate / paracingulate gyri, BA 32 (551)	16 out of 16
					L superior frontal gyrus, medial, BA 32 (501)	
					L anterior cingulate / paracingulate gyri, BA 32 (611)	
					R median cingulate / paracingulate gyri, BA 32 (266)	
					L median cingulate / paracingulate gyri, BA 24 (166)	
					R superior frontal gyrus, medial, BA 9 (116)	
R inferior frontal gyrus	50, 32, −2	−2.244	0.003	59	R inferior frontal gyrus, BA 45 (59)	14 out of 16

*MNI, montreal neurological institute; HE, hepatic encephalopathy; R, right; BA, brodmann area; L, left.*

### Subgroup Meta-Analysis of Studies Including Patients With Non-hepatic Encephalopathy

A total of seven studies, including patients with NHE, as a subgroup were analyzed, including seven sets of data, 153 patients with NHE, and 167 healthy controls (Table 1). Subgroup analysis showed GMV reduced in the cerebellar, the bilateral basal ganglia, and the insula compared with the control group. Furthermore, the volume of gray matter in the bilateral thalamus and the calcarine fissure and its surrounding cortex increased compared with the control group (Table 4 and Figure 2).

**TABLE 4:** Altered gray matter volume for subgroup meta-analyses between patients with NHE and healthy controls.

	Maximum			Cluster		
Brain regions	MNI coordinates, x, y, z	SDM value	*P*-value	No. of voxels	Breakdown (no. of voxels)	Jackknife sensitivity analysis (combination of studies detecting the differences)
**NHE > control**						
L thalamus	−4, −24, 8	2.920	<0.001	830	L thalamus (431)	7 out of 7
					R thalamus (399)	
L calcarine fissure / surrounding cortex	−4, −64, 8	1.461	<0.001	457	L calcarine fissure / surrounding cortex, BA 17 (176)	7 out of 7
					L lingual gyrus, BA 17 (131)	
					R lingual gyrus, BA 17 (75)	
					L precuneus, BA 23 (48)	
					R calcarine fissure / surrounding cortex, BA 17 (27)	
**NHE < control**						
R striatum	18, 10, 0	−2.435	0.001	1225	R striatum (498)	7 out of 7
					R lenticular nucleus, putamen (459)	
					R amygdala, BA 34 (158)	
					R insula, BA 48 (49)	
					R caudate nucleus, BA 25 (61)	
L striatum	−26, 6, 2	−2.269	<0.001	998	L striatum (397)	7 out of 7
					L lenticular nucleus, putamen, BA 48 (315)	
					L amygdala, BA 34 (174)	
					L insula, BA 48 (63)	
					L caudate nucleus, BA 25 (49)	
L cerebellum, hemispheric lobule	−8, −62, −38	−2.590	0.001	558	L cerebellum, hemispheric lobule (272)	7 out of 7
					Cerebellum, vermic lobule (266)	
					R cerebellum, hemispheric lobule (20)	

*MNI, montreal neurological institute; SDM, seed-based d mapping; NHE, non-hepatic encephalopathy; BA, Brodmann area; R, right; L, left.*

### Subgroup Meta-Analysis of Studies Including Patients With Minimal Hepatic Encephalopathy

All the patients with MHE were analyzed, including four sets of data, 79 patients, and 122 healthy controls (Table 1). The study showed GMV reduced in five brain regions, namely, the bilateral basal ganglia, the insula, the cerebellar hemisphere and the cerebellar vermis, the bilateral anterior cingulate gyrus extended to the right inferior frontal gyrus, and the superior temporal gyrus. GMV of patients with MHE increased in the bilateral thalamus, the right lingual gyrus, and the right calcarine fissure (Table 5 and Figure 2).

**TABLE 5:** Altered gray matter volume for subgroup meta-analyses between patients with MHE and healthy controls.

	Maximum			Cluster		
Brain regions	MNI coordinates, x, y, z	SDM value	*P*-value	No. of voxels	Breakdown (no. of voxels)	Jackknife sensitivity analysis (combination of studies detecting the differences)
**MHE > control**						
L thalamus	−14, −28, 10	3.521	<0.001	885	L thalamus (406)	4 out of 4
					R thalamus (423)	
					L hippocampus, BA 27 (48)	
					R hippocampus (8)	
R lingual gyrus	8, −62, −2	1.081	<0.001	282	R lingual gyrus, BA 18 (237)	4 out of 4
					R calcarine fissure / surrounding cortex, BA 17 (45)	
**MHE < control**						
L cerebellum, hemispheric lobule	−10, −60, −22	−2.676	<0.001	1703	L cerebellum, hemispheric lobule (877)	4 out of 4
					Cerebellum, vermic lobule (576)	
					R cerebellum, hemispheric lobule, BA 19 (250)	
R striatum	20, 2, −4	−3.120	<0.001	1494	R lenticular nucleus, putamen, BA 48 (447)	4 out of 4
					R striatum (384)	
					R amygdala, BA 34 (160)	
					R caudate nucleus (192)	
					R parahippocampal gyrus, BA 28 (158)	
					R temporal pole, superior temporal gyrus, BA 38 (109)	
					R insula, BA 48 (44)	
L insula	−30, 10, 10	−2.676	<0.001	1465	L insula, BA 48 (596)	4 out of 4
					L striatum (286)	
					L lenticular nucleus, putamen, BA 48 (334)	
					L amygdala, BA 34 (189)	
					L parahippocampal gyrus, BA 28 (60)	
R anterior cingulate / paracingulate gyri	8, 32, 22	−2.701	<0.001	947	R anterior cingulate / paracingulate gyri, BA 32 (361)	4 out of 4
					L anterior cingulate / paracingulate gyri, BA 24 (398)	
					L superior frontal gyrus, medial, BA 32 (188)	
R inferior frontal gyrus	46, 44, 0	−2.937	0.001	499	R inferior frontal gyrus, BA 45 (499)	3 out of 4
R superior temporal gyrus	52, −16, 4	−2.467	0.001	327	R superior temporal gyrus, BA 48 (247)	3 out of 4
					R heschl gyrus, BA 48 (80)	

*MNI, montreal neurological institute; SDM, seed-based d mapping; MHE, minimal hepatic encephalopathy; BA, brodmann area; R, right; L, left.*

### Subgroup Meta-Analysis of Studies Including Patients With Overt Hepatic Encephalopathy

A total of four studies with patients with OHE were analyzed, including five sets of data comparing 101 patients and 140 healthy controls (Table 1). The subgroup analysis identified GMV reduction in the bilateral insula, the bilateral basal ganglia, the bilateral cingulate gyrus, and the cerebellum. GMV increased in the bilateral thalamus, the lingual gyrus, and the calcaneal fissure (Table 6 and Figure 2).

**TABLE 6:** Altered gray matter volume for subgroup meta-analyses between patients with OHE and healthy controls.

	Maximum			Cluster		
Brain regions	MNI coordinates, x, y, z	SDM value	*P*-value	No. of voxels	Breakdown (no. of voxels)	Jackknife sensitivity analysis (combination of studies detecting the differences)
**OHE > control**						
L thalamus	−8, −24, 4	8.224	<0.001	950	R thalamus (433)	5 out of 5
					L thalamus (424)	
					R lingual gyrus, BA 27 (93)	
L calcarine fissure / surrounding cortex	−14, −74, 0	1.192	0.001	252	L calcarine fissure / surrounding cortex, BA 17 (76)	5 out of 5
					L lingual gyrus, BA 18 (66)	
					R lingual gyrus, BA 18 (60)	
					R calcarine fissure / surrounding cortex, BA 17 (50)	
**OHE < control**						
L insula, BA 48	−28, 12, 6	−3.764	<0.001	3058	L insula, BA 48 (679)	5 out of 5
					L striatum (490)	
					R striatum (422)	
					R insula, BA 48 (388)	
					L lenticular nucleus, putamen, BA 48 (335)	
					R lenticular nucleus, putamen, BA 48 (309)	
					R amygdala, BA 34 (216)	
					L caudate nucleus (97)	
					R caudate nucleus (66)	
					L amygdala, BA 34 (56)	
L cerebellum, hemispheric lobule	−10, −60, −22	−2.676	<0.001	2008	L cerebellum, hemispheric lobule (967)	5 out of 5
					R cerebellum, hemispheric lobule, BA 19 (787)	
					Cerebellum, vermic lobule (254)	
R anterior cingulate / paracingulate gyri	0, 40, 28	−5.181	0.003	1141	R anterior cingulate / paracingulate gyri, BA 32 (508)	5 out of 5
					L anterior cingulate / paracingulate gyri, BA 24 (398)	
					L superior frontal gyrus, BA 32 (390)	
					R median cingulate / paracingulate gyri, BA 32 (145)	

*MNI, montreal neurological institute; SDM, seed-based d mapping; OHE, overt hepatic encephalopathy; BA, brodmann area; R, right; L, left.*

### Sensitivity Analysis

According to Table 3, the GMV alteration of the right thalamus, the right calcarine fissure and its surrounding cortex, the left insula, the cerebellum, and the right anterior cingulate gyri were highly replicable for the reason that they were kept throughout all of the 16 combinations of studies. The sensitivity analysis showed that theGMV decrease in the right inferior frontal gyrus were also significant in all but two combinations of the datasets.

Whole-brain jack-knife sensitivity analyses of the subgroup meta-analysis of studies involving patients with NHE show that the left thalamus, the left calcarine fissure and its surrounding cortex, the bilateral striatum, and the cerebellum were highly replicable, being preserved through all of the 7 combinations of studies (Table 4). In patients with MHE, the altered GMV in the left thalamus, the right lingual gyrus, the cerebellum, the right striatum, the left insula, and the right anterior cingulate were highly replicable, as they were kept throughout all of the 4 combinations of studies (Table 5). As for OHE, altered GMV in the left thalamus, left calcareous clef and peripheral cortex, left insula, cerebellum, and right anterior cingulate were highly reproducible because these findings were kept in all the five dataset combinations (Table 6).

### Meta-Regression

**FIGURE 3:**
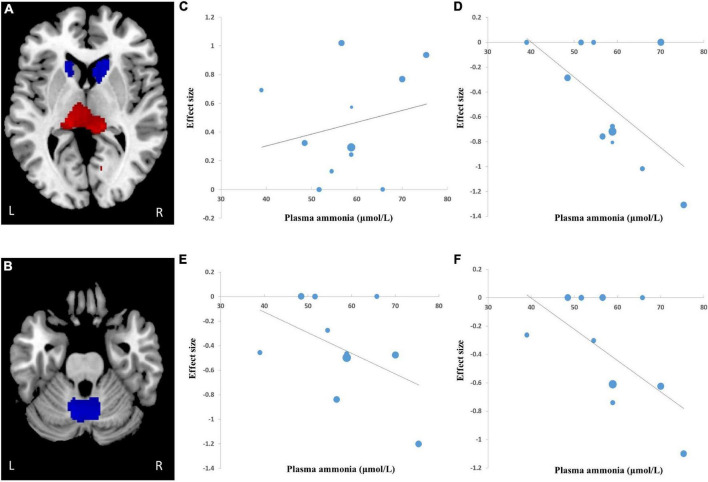
Results of meta-regression analysis of studies of patients with HE compared with healthy controls. **(A,B)** The regions of decreased (blue) and increased (red) gray matter with changes in plasma ammonia concentration. **(C)** The plasma ammonia concentration of patients with HE was positively associated with gray matter volume (GMV) in the left thalamus. The plasma ammonia of patients with HE was negatively associated with GMV in the cerebellum **(D)**, the right striatum **(E),** and the left striatum **(F)**. In the figure, the effect size required to create this plot has been extracted from the peak with the maximum slope significance, and each study is represented by a point whose size reflects the sample size; L, left; R, right.

A meta-regression analysis was used to analyze the association between plasma ammonia and GMV alteration in patients with HE. These variables were available for all the 228 participants in the 11 datasets. The plasma ammonia level positively correlated with the GMV in the left thalamic infract and negatively correlated with the GMV in the cerebellum and the bilateral striatum (Figure 3).

## Discussion

To the best of our knowledge, this is the first study to analyze the changes in brain GMV of patients with HE with SDM software and three subgroup analyses. Both the comprehensive analysis and the subgroup analysis have confirmed that patients with HE had extensive symmetry changes in gray matter. Our research may help to understand the potential neurodegenerative process of HE. An important finding of subgroup analysis is that, in patients with different degrees of HE, the changing area of brain GMV is similar, and the range of symptoms increases with the aggravation.

The current voxel meta-analysis mainly showed that GMV increased in the bilateral thalamus, the bilateral hippocampus, and the bilateral lingual gyrus extending to the calcarine fissure and the precuneus. In addition, the GMV decreased in the bilateral insula, the basal ganglia, the anterior cingulate gyrus extending to the superior frontal gyrus, and the cerebellum. The results of the sensitivity analysis remained largely unchanged and the results were robust and highly reproducible.

Several neuroimaging findings suggested that changes in the cortico-striato-thalamic pathway could play a crucial role in HE (Qi et al., 2012, 2013b,^2015^). As information from the cortex is returned to the thalamus indirectly or directly through the striatum—white matter system, the thalamus may act as a filter for sensory input (Nakajima et al., 2019). The basal ganglia are considered to be playing an important role in the pathophysiology of HE (Qi et al., 2013a). In previous studies, basal ganglia indicated functional disconnection with other brain regions and abnormalities in metabolism (Sharma et al., 2010; Chen et al., 2012), which resulted in the disinhibition of the thalamic activity and inputs more inhibitory information into the cortex, leading to neurocognitive dysfunction (Grillner and Robertson, 2015; He et al., 2020). In addition, manganese is known to deposit within intracranial structures, mainly the globus pallidus and the pituitary gland (Dietemann et al., 1998; Rovira et al., 2008). Previous studies have shown that manganese deposition is also one of the reasons for the loss of basal ganglia neurons (Kano and Morishima, 2021). Moreover, manganese can colocalize in such intracranial structures with other elements, such as iron or gadolinium. These elements showed cellular toxicity in neurons, which may also be the reason for the reduction of GMV in basal ganglia (Mallio et al., 2020). An increase in thalamic volume may indicate hypertrophy or hyperplasia of neurons or glia. It is speculated that the increase of thalamic volume is a compensatory effect of basal ganglia dysfunction, but the increase of thalamic volume cannot completely improve brain function (He et al., 2020).

The results showed that patients with HE also suffered from obvious increase in some brain regions, namely, the bilateral precuneus, the lingual gyrus, and the calcarine fissure and its surrounding cortex. Notably, these areas were linked to the visual functions (Rao et al., 2007; Rita Machado et al., 2017), which are relative to impaired visual information processing in patients with cirrhosis. The increase in the cortical thickness observed in patients with cirrhosis seems to contradict previous works of research (Montoliu et al., 2012; Wernberg et al., 2019). The reason for such inconsistency remains unclear. However, a previous study’s results were similar to ours, which speculated that the increase of cortical thickness was related to mild brain edema in patients with HE (Wu et al., 2015). With the progression of the disease, the scope of the increase in the volume of related brain regions is reduced. We speculated that the progressive atrophy of the cortex caused by dysfunction weakens the increase in the GMV. The related neuropathological mechanism is not clear and is worthy of further study.

Our study found that, in comparison with the healthy control group, patients with HE had wider GMV reduction areas with the progress of the disease. These findings have been shown in previous studies (Garcia-Martinez et al., 2011; Lin et al., 2019). The bilateral insula is the most significant area of GMV reduction, the decreased insula volume has been revealed in HE, which correlates with cognitive alterations (Chen et al., 2017; García-García et al., 2017). It is found that the resting-state neuronal activity represents abnormal regional uniformity in the insula of patients with HE (Ni et al., 2012; Chen et al., 2016). The insula integrates internal and external stimuli and plays an important role in episodic memory processing. The anterior cingulate cortex is a significant region for attentional control, response suppression, and error detection (Kerns et al., 2004). The anterior cingulate cortex and the insula participate in the detection and the location of external stimuli and internal events (Flook et al., 2020) and is also a key brain region of the default mode network (DMN; Zhang et al., 2013; Wertz et al., 2020). Studies have shown that DMN has high metabolic activity at rest. Therefore, decreased gray matter voxel of the anterior cingulate cortex may be the neurobiological basis of cognitive impairment. In addition, we also noted that, without HE, the volume of the anterior cingulate cortex region did not change in patients with cirrhosis, which maybe because of the early stage of the disease.

The cerebellum is a regulator of motor function, but a link between cerebellum changes and impaired executive function has been established (Kühn et al., 2012). Our results showed that the area of reduced cerebellar volume increased significantly with the progression of the disease. Studies have shown that the cerebellum was more susceptible to the harmful effects of hyperammonemia than the cerebral cortex (Rodrigo et al., 2010). Our study also found that the volume of the bilateral striatum and the thalamus changed with the increase of plasma ammonia, which could be caused by an increased blood flow of related brain regions in patients with cirrhosis (Felipo et al., 2014). Cerebral blood flow in patients with HE has regionally selective changes, such as decreased blood flow to cortical regions but significantly increased blood flow to the basal ganglia, the cerebellum, and the thalamic structures (Butterworth, 2019). These regions are more susceptible to plasma ammonia. New animal models using ammonia as a precipitation factor also confirmed cerebellar neuron loss (García-Lezana et al., 2017). Gray matter loss reflects ammonia-related neurological changes.

The discovery and measurement of HE are challenging in clinical practice. Nowadays, artificial intelligence and machine learning have been increasingly applied to disease diagnosis, especially medical image recognition (Supekar et al., 2022). We reviewed previous articles and obtained a relatively unified result of GMV change in patients with HE, which provides a basis for delineating ROIs in machine learning. EEG is a common tool for monitoring HE (Amodio and Montagnese, 2015). However, EEG signals may be damaged by background noise, which leads to inconsistent diagnostic accuracy (Mutanen et al., 2018). Machine learning algorithms can analyze records containing a large number of variables and can find complex linear or non-linear relationships between variables (Gazda et al., 2021). Machine learning and deep learning methods have been used to analyze EEG to predict various chronic psychiatric illnesses (Natu et al., 2022; Paul et al., 2022). Using artificial intelligence and machine learning to comprehensively analyze MRI and EEG to diagnose HE more accurately may be the direction of future research.

This study still has some limitations. First, due to the sample size, the power of the results of this study may be limited, which is similar to the early voxel-based meta-analysis. Second, there are still some deviations in the VBM method, especially in areas with large anatomical variation; it may excessively show population differences, which is due to its relative insensitivity to more spatial changes (Bookstein, 2001; Tisserand et al., 2002). Another issue is that the meta-analysis is related to coordinates in published studies, leading to less accurate results (Zhou et al., 2014). Moreover, only 9 studies met the inclusion criteria for the quantitative analysis in our research. A small number of studies would reduce the credibility of the results, especially the subgroup analysis should be interpreted with caution as they were driven by less than 10 studies. In addition, the lack of a protocol with *a priori* methods, the inclusion of English language papers only, the absence of searches for gray literature, and the use of a non-validated quality assessment checklist may have introduced a range of biases.

## Conclusion

Based on the available imaging works of literature, this meta-analysis revealed widespread and significant changes in GMV in patients with HE. The subgroup analysis suggested that, in patients with different degrees of HE, the changing area of GMV was similar and the range of symptoms increased with aggravation. The change of GMV in patients with HE was significantly related to plasma ammonia.

## Data Availability Statement

The raw data supporting the conclusions of this article will be made available by the authors, without undue reservation.

## Author Contributions

JL, JW, and CZ: conceptualization, methodology, and project administration. LZ, WZ, and LC: investigation. TS and YR: resources and data curation. LZ and YC: writing—original. BS and LC: draft, formal analysis, and visualization. All authors contributed to the article and approved the submitted version.

## Conflict of Interest

The authors declare that the research was conducted in the absence of any commercial or financial relationships that could be construed as a potential conflict of interest.

## Publisher’s Note

All claims expressed in this article are solely those of the authors and do not necessarily represent those of their affiliated organizations, or those of the publisher, the editors and the reviewers. Any product that may be evaluated in this article, or claim that may be made by its manufacturer, is not guaranteed or endorsed by the publisher.
